# Effect of Ceramic Thickness and Adhesive Light Curing on Bond Strength of Resin Cements to Enamel

**DOI:** 10.1055/s-0045-1804888

**Published:** 2025-04-23

**Authors:** Hillary O. de Alvarenga, Kusai Baroudi, Raghavendra M. Shetty, Elias D. Berdouses, Marianna Pires de Oliveira, Anna Laura Diniz, Gabriel Ferreira, Laís Regiane Silva-Concilio, Marina Amaral

**Affiliations:** 1 Department of Dentistry, University of Taubaté – UNITAU, Taubaté, SP, Brazil; 2 Department of Clinical Sciences, College of Dentistry, Ajman University, Ajman, United Arab Emirates; 3 Centre of Medical and Bio-allied Health Sciences Research, Ajman, United Arab Emirates

**Keywords:** laminate veneers, cementation, shear strength

## Abstract

**Objectives:**

For cementation of ceramic restorations, a layer of adhesive followed by resin cement is applied to the treated enamel surface. The light activation of adhesive may occur before or simultaneously with the resin cement. The aim of this study was to evaluate the influence of ceramic thickness and previous light activation of adhesive on shear strength of resin cement to enamel.

**Materials and Methods:**

Vestibular bovine enamel was bonded to lithium disilicate ceramic cylinders with resin cement. The samples were divided into two groups, according to the ceramic thickness (1 or 2 mm). The cylinders had one surface treated for cementation and the enamel surface was treated with acid etching and adhesive system. Only half of samples received light activation of the adhesive prior to cementation. The samples were stored for 30 days in water at 37°C, and then subjected to the shear bond strength test.

**Statistical Analysis:**

Two-way analysis of variance was applied to evaluate the influence of previous light activation and ceramic thickness on the bond strength to enamel ( *α* = 0.05).

**Results:**

The results of this study indicated that there is no significant difference in the shear adhesive strength between ceramics and dental enamel in relation to the factors evaluated.

**Conclusion:**

It is concluded that bond strength is not affected by neither the previous adhesive light activation nor ceramic thickness (1 or 2 mm).

## Introduction

Restorative dentistry is constantly seeking innovative solutions to restore esthetics and functionality of previously compromised teeth. Among the available techniques and materials, ceramic restorations have become an ideal aesthetic treatment for anterior restorations. 1 These restorations, known for their ability to precisely mimic the color, texture, and translucency of tooth enamel, have gained prominence as an efficient alternative for the restoration of damaged anterior and posterior teeth. 2 The growing preference for ceramic restorations is also largely due to their ability to preserve tooth structure, requiring minimal wear compared with other restorative procedures such as metal ceramic crowns. Furthermore, the glass ceramics have remarkable adhesive properties, contributing to reliable and long-lasting adhesion. 3 To achieve effective adhesion between ceramic laminates and tooth enamel, an adhesive cementation protocol needs to be followed meticulously. This protocol commonly involves etching the enamel with 37% phosphoric acid for 15 or 30 seconds followed by adhesive application. On the ceramic surface, the ceramic etching is performed with 5 or 10% hydrofluoric acid for 20 to 120 seconds depending on the ceramic type, followed by the application of silane. Resin cements are used to bond both surfaces as they adhere to the tooth structure and have acceptable aesthetics, high mechanical resistance, and are insoluble in the oral environment 1 ; however, it is also important to consider the degree of conversion of resin cement, which changes the thickness of the ceramic. 4

The success of ceramic restorations is determined, among other factors, by the strength and durability of the adhesion between the substrates and the bonding agent involved. 2 3 The adhesive luting is essential for retention of partial coverage restorations, including laminate veneers, and combines mechanical and micromechanical retentions and chemical and molecular bonding mechanisms. 5 The adhesive cementation is focused in esthetic, strength, and durable retention. 6 The adhesive is a fundamental element in this process. Resin cements associated to etch-and-rinse adhesives proved to provide higher bond strength, especially on enamel surfaces, 7 and the photoactivation of adhesives plays a crucial role in the formation of a stable bond between the resin cement and tooth enamel.

The light activation of adhesive can be performed in two different moments: after application on tooth surface before applying the resin cement, or simultaneously with the light activation of resin cement. When the adhesive is photoactivated before cementation, there is a risk of formation of a thick layer of adhesive, leading to misfit of the restoration, due to incorrect positioning of the restoration. This may result in marginal misfit of restoration, occlusal interference, and inappropriate support of restoration by substrate—all characteristic related to clinical failures as marginal pigmentation, restoration fracture due to clinical adjustments, and decreased occlusal thickness.

On the other hand, if light activation occurs concomitantly with resin cement, there is a risk of unsatisfactory polymerization of the adhesive, due to the attenuation of light from light curing unit when passing through ceramic restoration and resin cement before reaching the adhesive, resulting in a poor hybrid layer. 8 9 10 A lower degree of conversion of the adhesive may also occur, and decrease adhesive mechanical strength, biocompatibility, and increase permeability as disadvantages. 11 12

A low degree of conversion of cement and/or adhesive may lead to color changes, toxicity from the residual (unreacted) monomer, decreased adhesion, and postoperative sensitivity, increasing the risk of nanoinfiltration, cavities, and ceramic fractures. 13 14 15 16 On the other hand, the concomitant light activation of adhesive and composite may benefit from the elimination of an oxygen-inhibited layer, which is formed with the photoactivation of adhesive and may improve bond strength. 17

Thus, the aim of this study was to evaluate the influence of ceramic thickness and light activation of adhesive on shear strength of resin cement to enamel.

## Materials and Methods

In the present *in vitro* experimental study, 20 bovine teeth were used, coming from a certified slaughter house, exempt from approval by the research ethics committee, according to the Arauca law (n° 11,794, 10/08/2008). The crowns were separated from the roots by sectioning at the cementoenamel junction with a diamond cutting disc attached to the handpiece. Crowns were embedded in chemically activated acrylic resin, into a 25 mm diameter × 15 mm polyvinyl chloride matrix, with the vestibular enamel exposed on the surface. Exposed enamel received an initial polishing with 600 grit water sandpaper, coupled to a bench polisher (Aropol E, Arotec, Cotia, Brazil). Enamel surface was acid etched with 37% phosphoric acid (CONDAC 37, FGM) for 30 seconds followed by cleaning with water and drying with air.

Lithium disilicate ceramic cylinders (IPS e.max CAD, Ivoclar Vivadent) were sectioned from a green block, with a diamonded trephine, with 2.4 mm in diameter, with two different thicknesses: 1 and 2 mm. Ceramic cylinders were then crystalized in specific furnace (850°C/1 minute). The ceramic cylinders had one of the surfaces treated with the application of 5% hydrofluoric acid for 20 seconds followed by cleaning with water and drying with air, and application of silane agent (Prosil, FGM).

A layer of light cure conventional adhesive (Ambar APS, FGM) was applied on the enamel surface, had solvent evaporated with gentle air blasting, and 20 samples received light activation of the adhesive for 20 seconds (Bluephase, Ivoclar Vivadent), while the other 20 samples had adhesive and not light activated. Immediately afterwards, a ceramic cylinder of thickness 1 and 2 mm was cemented to each crown with light-activated resin cement (Variolink N, Ivoclar Vivadent) onto the enamel surface (two cylinders per tooth).

To cement the cylinders on the enamel surface, a blackout cardboard shield was used, with a 2.5-m thick hole to limit the passage of light from the curing unit through the ceramic cylinder, not reaching the adhesive interface directly. Light activation was performed for 30 seconds on each ceramic cylinder (Bluephase, Ivoclar Vivadent).

Four groups were formed: (1) 1 mm ceramic thickness with previous light activation of adhesive, (2) 1 mm ceramic thickness without previous light activation of adhesive, (3) 2 mm ceramic thickness with previous light activation of adhesive, and (4) 2 mm ceramic thickness without previous light activation of adhesive.

The samples were stored for 30 days into 37°C distilled water, and then subjected to the shear bond strength test. Samples were positioned in a universal testing machine (MBio, BioPDI), with the adhesive interface perpendicular to the ground, and a chisel applied an increasing load, parallel to the adhesive interface until the sample fractured. The maximum load was recorded and the Shear Bond Strength (SBS, MPa) was calculated as a function of the adhesive interface area (A, mm ^2^ ) and the maximum applied load (L, N): SBS = F/A. An average bond strength was calculated for each group tested ( *n* = 10).

Data distribution was assessed using the Kolmogorov–Smirnov normality test. Two-way analysis of variance was applied to evaluate the influence of previous light activation and ceramic thickness on the bond strength to the enamel ( *α* = 0.05).

Failure analysis was performed under stereomicroscope (Discovery V20, Carl Zeiss) with 100× magnification and classified as (1) adhesive failure between adhesive and enamel, (2) cohesive failure of ceramic, (3) cohesive failure of enamel, (4) cohesive failure of resin cement, and (5) mixed failure—association of adhesive with cohesive failure.

## Results

**Fig. 1:**
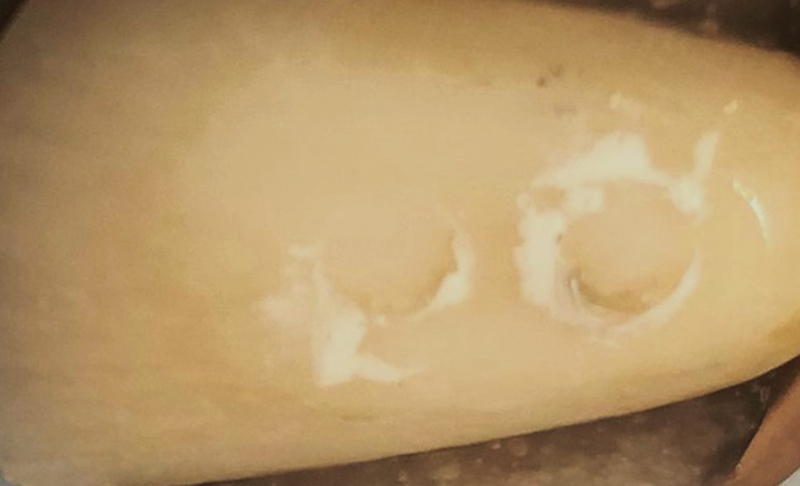
Image of enamel surface after shear bond strength test, representing adhesive failure. Shear load was applied to the interface between ceramic cylinder and enamel surface, from top to bottom of the image.

None of the factors evaluated (previous light activation: *p* = 0.288 or ceramic thickness: *p* = 0.786) affected the shear adhesive strength between ceramic and tooth enamel. Table 1 shows the mean values and standard deviation for bond strength for each group. All failures found were classified as adhesive between resin cement and enamel ( Fig. 1 ).

**Table 1:** Means (MPa) and standard deviation of adhesive strength for each group tested

Previous light activation of adhesive	Ceramic thickness		Total
	1 mm	2 mm	
With	9.76 (9.28)	16.65 (5.15)	13.2 (8.1)
Without	18.7 (9.82)	13.3 (8.40)	16.2 (9.3)
Total	14.3 (10.4)	15.0 (6.9)	

## Discussion

The results showed that, for ceramics up to 2 mm thick, the absence of prior light activation of the adhesive did not affect the bond strength values for glass ceramic cemented to tooth enamel with light-activated resin cements. The influence of ceramic thickness in bond strength is not a concern. It is stated that the thickness of ceramic did not influence the union or the durability of ceramic, 18 but the degree of resin cement conversion is affected in thicker ceramics was already reported. 19 Also, the degree of resin cement conversion may depend on the thickness of the ceramic when it is thicker than 1.5 mm. 20 For composite resins, the degree of conversion of adhesive was affected only under 4 mm restorations, and it could be improved anaerobically. 17

Several types of resin cements are used for cementing ceramic-laminated veneers and many dentists prefer light-cured cements as they enable better control of the cement through the use of light curing. 1 The results of this study indicated that there is no significant difference in the shear adhesive strength between ceramics and dental enamel when using 1 and 2 mm ceramics ( Table 1 ). It is possible that the thickness of the ceramic does not affect the passage of light for the photoactivation of the resin and adhesive cement. Thicker restorations (i.e., 4 mm) may affect the degree of conversion of adhesives and decrease bond strength. 17 Improved bonding strategies of cementation must be evaluated for thick restorations, but they will probably not be applied to laminate veneers, since in such restorations, the thickness of the restorative material is reduced. The present study applied one shade and opacity of ceramic and resin cement, and results may vary in different conditions. 21 The opacity of ceramic/cement may interact with ceramic thickness and decrease light delivery in significant levels, resulting in inappropriate adhesive conversion.

The literature mentions that the polymerization of adhesive prior to the cementation of ceramic pieces can cause misfit of the prosthetic piece, as it will form a thick layer of polymerized adhesive. In the concomitant polymerization of adhesive and cement, there is the possibility of defective polymerization of the adhesive, which may affect the formation of hybrid layer, given that the light from the photopolymer can be blocked by the resin cement and ceramic layers. 8 22 Unsatisfactory photopolymerization can result in color changes, toxicity, lower adhesion, and decreased mechanical properties of composite materials. 23 24 25

Besides not affecting the bond strength values, the degree of conversion of adhesive and/or resin cement may have been affected, since correlation may not exist between the degree of conversion and the shear bond strength values. 26 Prior photoactivation of the adhesive guaranteed satisfactory results, when the mechanical properties of the adhesive was evaluated. 27 Color, stability, and mechanical properties of resin cement were not influenced by different photopolymers. 8 Meanwhile, prior photopolymerization of the adhesive was reported not to affect color stability. 10

Color changes are the main reason for replacing aesthetic restorations, 1 therefore, resin cement must have long-term color stability to guarantee acceptable results. Cement discoloration can occur due to some extrinsic and intrinsic factors. The intrinsic factors responsible for the discoloration of ceramic restorations are mainly related to the properties of the resin cement, such as its chemical composition (light cure, type of filler, matrix composition), type of polymerization, conversion rate, and presence of nonreactive monomers.

One advantage of not performing adhesive activation previously to cement application is to avoid oxygen-inhibited layer, which may decrease the degree of conversion at adhesive–resin interface. 28 The oxygen-inhibited layer was first considered necessary for adding a subsequent resinous material, as composite resin or resin cement. However, studies may demonstrate good, not relevant of bad effects of oxygen-inhibited layer. 29 30 Low conversion rates at adhesive–resin interface may result in lower bond strength. Thus, the prevention of oxygen-inhibited layer formation in the present study may have corroborated for no difference in bond strength when adhesive was not activated prior to cement application.

Regarding the adhesive protocol used in this study, preconditioning with phosphoric acid on the enamel surface is necessary for effective adhesion. 31 Etching enamel with phosphoric acid is still the most reliable method for obtaining more durable and sealed restorations. 32 Adhesion to enamel is more favorable than adhesion to dentin, as enamel is a more mineralized structure. This fact may have contributed to similar values of adhesive strength between the groups.

As shown by failure analysis, besides more reliable than adhesion to dentin surface, all failures occurred at resin cement–enamel surface. Adhesion to glass ceramic surface is well established by acid etching and silane application. The adhesion to tooth tissues is still more challenging than adhesion to ceramic surface, requiring more studies and improvement.

## Conclusion

Given the results of this research, it can be concluded that neither the previous photoactivation of the adhesive nor the thickness of the material affected the adhesive resistance between tooth enamel and lithium disilicate up to 2 mm thickness.
